# Participatory systems science for enhancing health and wellbeing in the Indian Ocean territories

**DOI:** 10.3389/fpubh.2023.1013869

**Published:** 2023-06-15

**Authors:** Steven Allender, Syarifah Liza Munira, Siobhan Bourke, Emily Lancsar

**Affiliations:** ^1^Global Centre for Preventive Health and Nutrition, Institute for Health Transformation, Deakin University, Geelong, VIC, Australia; ^2^Department of Health Services, Research and Policy, National Centre for Epidemiology and Population Health, Australian National University, Canberra, ACT, Australia

**Keywords:** diabetes, systems thinking, health and wellbeing, Indian Ocean territories, obesity

## Abstract

**Objectives:**

Co-creation of diabetes and obesity prevention with remote communities allows local contextual factors to be included in the design, delivery, and evaluation of disease prevention efforts. The Indian Ocean Territories (IOT) comprise the Christmas (CI) and Cocos Keeling Islands (CKI) and are remote Australian external territories located northwest of the mainland. We present results of a co-design process conducted with residents of IOT using realist inquiry and system mapping.

**Methods:**

Interviews with 33 community members (17 CI, 14 CKI, 2 off Islands) on causes and outcomes of diabetes (2020/21) comprising community representatives, health services staff, dietitians, school principals and government administrators. Interviews were used to create causal loop diagrams representing the causes of diabetes in the IOT. These diagrams were used in a participatory process to identify existing actions to address diabetes, identify areas where more effort would be valuable in preventing diabetes, and to described and prioritize actions based on feasibility and likely impact.

**Findings:**

Interviews identified 31 separate variables categorized into four themes (structural, food, knowledge, physical activity). Using causa loop diagrams, community members developed 32 intervention ideas that included strengthening healthy behaviors like physical activity, improving access to healthy and culturally appropriate foods, and overcoming the significant cost and availability limitations imposed by remoteness and freight costs. Interventions included relatively unique Island issues (e.g., freight costs, limited delivery timing), barriers to healthy food (e.g., limited fresh food availability), physical activity (e.g., transient workforce) and knowledge (e.g., multiple cultural backgrounds and language barriers, intergenerational knowledge).

## 1. Introduction and background

Diabetes is a major chronic condition impacting health systems (1). Type 2 diabetes mellitus (T2DM) is the most common type of diabetes, and is associated with multiple risk factors including age, family history, and ethnicity. Other risk factors include modifiable lifestyle characteristics including physical activity, nutrition, smoking, and weight management. People with diabetes have a higher risk of developing high blood pressure, heart disease, stroke, kidney failure, as well as circulation problems, nerve damage, lower limb amputations and impaired vision (2).

Remote and very remote areas in Australia (3) experience a disproportionately higher rate of diabetes (6%) compared with major cities (4%) (4). Communities in the Indian Ocean Territory, one of Australia’s most remote areas, experience even higher rates, at 8 and 11%, respectively, for Christmas Island (CI) and Cocos (Keeling) Island (CKI). The proportion of community members living with overweight and obesity is 84% on CKI and 77% on CI, higher than regional and remote (69%) and mainland Australians (65%) (5). Obesity increases the risk of diabetes (6) and two main factors that contribute to obesity: poor diet and inadequate physical activity, are heightened for the communities in the IOT (5). As one example, the cost of food, and particularly fresh fruits and vegetables, is estimated to be 81.1% (CKI) and 82.1% (CI) higher in the IOT than the nearest major Australian city, Perth (7). The high cost and limited availability of fresh food compared to packaged and processed foods serves as a disincentive for residents to eat healthy food.

Living in a rural and remote setting is a significant factor in disease risk profile and outcomes (8) relating to access to healthy foods (9) and structural elements, e.g., support for active modes of transport. Public health has moved from ignoring remoteness as a cause of disease to making it a key consideration in intervention design (10). Engaging with remote communities has provided unique insight into the drivers of ill health previously not possible in remote communities (11, 12). System science represents a suite of research methods that start with the aim of understanding the complexity of cause and effect in a given problem and using this understanding as the basis of planning a response (13). Approaches, like community-based system dynamics (14), provide methods to actively work with members of a community to build a clear picture of the different relationships of cause and effect creating a problem and understand how these relationships change and interact over time. The subsequent understanding of the system can be more comprehensive than traditional approaches and may include the status of the community, resources, and political acceptability of change, among others (15). Interventions built on these techniques are more suited to place than externally developed and non-consultative intervention strategies. These techniques have been used to support communities responding to climate change (16), COVID (17), GP prescribing behavior (18), and obesity (19, 20).

In this paper we report on a study which set out to:

- create a shared understanding of the drivers of diabetes on CI and CKI
- provide an overview of existing literature to community members and help them understand how interventions may work for people with diabetes in remote and culturally diverse communities
- develop a set of practical ideas to address some of these drivers
- identify policy settings and potential initiatives that would support these changes, and
- identify points in the system where a diabetes intervention could fruitfully be used.

The objective of this study was to co-create, with community members of the Christmas and Cocos Keeling Islands, a set of possible policy initiatives to prevent or manage diabetes in the Indian Ocean Territories.

## 2. Methods

### 2.1. Study design

This study is the second half of a two-part research which aimed to identify and develop possible policy initiatives to prevent/manage diabetes in the IOT. The first part was a realist review of the literature (21) which informed this paper including the systems mapping (22) which was further complemented by qualitative interviews with key stakeholders. Realist methods are used when the intent is to understand processes and mechanisms by which observed phenomena come to take their current shape.

Phase one: comprised a realist enquiry (in preparation) to expand relationships between context, mechanism and outcome in the IOT. Initial program theories emerged from interviews with key staff and community members. A literature search for these theories identified >150 studies which confirmed evidence for program theories including subsidizing fresh fruit and vegetables, sustainable farming, and engaging communities to improve health. These were further validated with a second round of interviews. The comprehensive description of this study is the subject of a separate manuscript.

Briefly, for the first set of interviews in the first part of the research, we adopted a constructivist grounded theory analysis approach, which involves a process of iterative data collection and constant comparative analysis of the raw data, the literature, and the research memo to help inform the research; ensuring that the analysis and findings are “grounded” in participants’ own words and experiences. The premise behind constructivist grounded theory is that “data do not provide a window on reality. Rather, the ‘discovered’ reality arises from the interactive process and its temporal, cultural, and structural contexts.” This approach is well-suited to investigate dynamic and complex public health challenges composed of distinctive yet interrelated issues that together form a complete picture of systemic issues in a community.

Data saturation was reached in the first phase of the research from interviews with key staff and community members in the Indian Ocean Territories, resulting in initial program theories, that were refined by a realist synthesis and further validated with a second round of interviews. The result of the first part informed this paper, in particular an initial causal loop diagram that was presented to key stakeholder and community members and discussed through a facilitated group model building process using a system mapping approach.

To ensure rigor, transcripts were analyzed by two researchers (SB and LM) with authenticity achieved through verbatim transcription and confirmation of this by listening to recordings. The researchers met daily during the data collection period, and afterwards met weekly to discuss and refine new concepts. Identified patterns were refined as new data was collected. The validity of the findings were enhanced by incorporating findings from debriefings into the subsequent interviews and into the analysis. Feedback from the wider research team was incorporated to establish credibility. Quotes from participants with a range of views further supported accurate interpretation and rigor.

Phase two: This study is the second half of a two-part research which aimed to identify and develop possible policy initiatives to prevent/manage diabetes in the IOT. The first part was a realist review of the literature which informed the second half of the research including the systems mapping that was further complemented by qualitative interviews with key stakeholders.

Findings from the first phase of the research identified seven theories that influence the success of the target initiatives, across the spectrum of the socioecological model in the IOT: (i) subsidies, (ii) hypothecated taxes, (iii) sustainable farming, (iv) engaging with community organizations and individuals to create a healthier IOT community, (v) engaging with food retailers on the island, (vi) culturally sensitive approach to care, (vii) empowering the community to become actors for change.

These theories were refined by searching the literature for empirical evidence and conducting qualitative interviews with IOT community members. In particular, the community consultations identified a real need for empowerment within the community through meaningful and impactful engagement. The understanding of these mechanisms and interactions translates into useful points for designing and understanding the success of interventions for diabetes especially in complex context as those observed in the IOT.

Interviews during the second phase of the research identified 31 separate variables categorized into four themes (structural, food, knowledge, physical activity). Using causal loop diagrams, community members developed 32 intervention ideas that included strengthening healthy behaviors like physical activity, improving access to healthy and culturally appropriate foods, and overcoming the significant cost and availability limitations imposed by remoteness and freight costs. Interventions included relatively unique Island issues (e.g., freight costs, limited delivery timing), barriers to healthy food (e.g., limited fresh food availability), physical activity (e.g., transient workforce) and knowledge (e.g., multiple cultural backgrounds and language barriers, intergenerational knowledge).

Causal loop diagrams were built to represent the logic of interview data. Community based participatory group model building techniques were used to review the logic model and develop action plans. We worked with residents of the IOT across all phases of the project, this co-production of the research can enhance rigor in qualitative research through the integration of diverse perspectives and interpretations (23). Rigor was further enhanced through using well established scripts for the participatory activities, which provide close detail on running of sessions and allow replicability (14, 22). The session began with the presentation of an evidence brief describing the issue of diabetes and obesity on the IOT, current knowledge about cause and prevention in the research literature. Specifically, participants were presented with the causal loop diagram and a presentation was given on how to understand the conventions of causal loop diagrams describing how the variables were identified as actions leading to, or resulting from, diabetes or obesity in IOT. We described how connections between these variables were identified and the direction of cause and effect captured with an arrow showing the relationship between each variable as either positive (solid line; as one variable changes the other changes in the same direction, or negative; as one variable changes the other changes in an opposite direction). Participants were then provided time to review the model, considering where it made sense, where detail was missing and what they would change or add to improve the model. Participants were then asked to identify and locate where on the map there was existing action, where further action was needed and where the participants felt they had power to act. Participants then developed as many actions as they could think of and prioritized these based on potential impact and feasibility, with emphasis on whether changes were single actions or engaged across several variable or engaged feedback loops (19).

### 2.2. Setting

The Indian Ocean Territories (IOT), comprising Christmas Island (CI), and the Cocos (Keeling) Islands (CKI), are located northwest of mainland Australia and are categorized as some of the most remote communities in the country (5). The population of the IOT was 2,387 (1,843 on CI and 544 based on CKI) in 2021 with economies centered on phosphate mining, the Immigration Detention Centre (Christmas Island) and tourism. Around one quarter of inhabitants are Caucasian with significant Malay, Chinese and Cocos Malay populations. In CKI, nearly two-thirds (63.6%) of households reported that a non-English language was spoken at home (24).

### 2.3. Theoretical framework

Phase one: Qualitative stakeholder interviews and synthesis of existing literature following a realist review method (25, 26) was used to build an understanding of the existing health system and constraints relating to the IOT in relation to evidence on diabetes prevention and management. A semi-structured topic guide was developed and informed by the findings from the initial identification of the program theories.

Phase two: Data collection and analysis were informed by community based participatory system dynamics and utilized group model building (15). The intention of these techniques was to surface the mental models of community members regarding the causes of diabetes for the IOT and couple this with the existing evidence base about the causes and prevention of diabetes to develop context specific understanding of potential intervention areas. The underlying theoretical framework posits that change is created where communities engage with and respond to the complexity of cause and effect in community based health problems. Our theory of change, presented in detail elsewhere (15), identifies how community change can engage multiple accumulating factors (known as stocks) changing in relation to balancing and reinforcing feedback loops. This theory of change describes the potential roles for people in leadership positions, their response to a community public health need, community involvement and subsequent quality of action amplified by community buy-in and tailoring of actions to the local context. Higher quality actions and community exposure to actions result in improved community health behaviors.

### 2.4. Participant recruitment

#### 2.4.1. Phase one

Participants included key stakeholders/policy makers in the IOT and general community members. Purposive sampling was used to recruit participants from IOT health services, education sector, and IOT administrators. Community members were identified through snowball sampling recommended by key stakeholders. Twenty IOT health service staff and 13 community participants were recruited. The IOTHS staff were phone interviewed by three research team members (SB, LM and EL). For the community member interviews, six face-to-face interviews were conducted by the community researchers (PM and AH) and seven telephone interviews were completed by the research team (LM and SB). Seventy percent of the IOTHS staff and 38% of the community interviewees were female. The IOTHS staff interviewees had been living on the island for 0.25–49 years, while community interviewees for 2–32 years.

#### 2.4.2. Phase two

Participants were recruited through advertisements translated to the local languages and placed at various locations on the islands and in the island-wide newsletter. This was followed up by the community researcher on each island, who assisted with recruitment and allowed for different opportunities for engagement with the community. Participants included stakeholders from council/shire, IOT administrator office, high schools, IOT health services, and representatives of community-based organizations, such as the health services community advisory committee, vocational training association, and community resource center. Purposive sampling was used to recruit 25 community members with understanding of diabetes in the IOT. Participants were 18 years or older and living in the IOT. Additional key informants were identified through snowball sampling.

### 2.5. Data collection and analysis

Phase one: data from the first set of qualitative interviews combined with a realist synthesis of the literature were used to generate seven initial program theories. These program theories were that obesity and diabetes would be reduced if it were possible to: (1) Improve access to fresh fruit and vegetables (subsidies); (2) Improve access to fresh fruit and veg (tax); (3) Improve access to fresh fruit and veg (sustainable farming); (4) Engaging with community organizations and individuals to create a healthier IOT community; (5) Engage with food retailers on the island; (6) Provide a culturally sensitive approach to care; and (7) Empower the community to co-design a locally relevant diabetes intervention. These initiatives were then confirmed, refined, and refuted through qualitative interviews with community members.

Phase two: the data collected in phase one were used to identify relationships of cause and effect relating to diabetes and obesity on the IOT. These relationships were entered into the *Systems Thinking in Community Knowledge Exchange* (STICKE) software^1^ (27) and the links between variables identifying cause and effect were created. These links were then coded to reflect whether a change in the causal variable led to a similar change in the resultant variable (i.e., increasing family activity increased exercise culture) or whether they changed in opposite directions (i.e., an increase in sustaining activity groups decreased sedentary behaviors). These maps were then coded to align with the codes identified in phase one, specifically identifying areas of the map referring to structural, food, knowledge, and physical activity elements. We collected data via qualitative structured group process using video conferencing facilities available through the training center on each island managed by Indian Ocean Group Training Association (IOGTA). The facilities at the training center included Wi-Fi connection, projectors, and a smartboard. They also had a landline access with speaker phones in case of disruption with the Wi-Fi connection. The original plan was to conduct these sessions in person, but travel restrictions due to COVID-19 meant we were unable to conduct these in person. In short, local health team members on each island were recruited and trained to support the delivery of the workshop sessions described below while the team facilitated the sessions remotely. The local facilitators were sent materials ahead of the sessions, notably system maps (A3 size), sticky dots (black and pink) sticky labels (blue and yellow), action ideas recording templates (A5 sheets). One session was conducted on CI and a second on CKI in September 2021 and November 2021. The session format is described in Table 1.

**Table 1:** Workshop format and data collection for phase two.

Agenda item	Time (mins)	Description
Welcome	10	The study lead introduced the session and the purpose of the study, welcomed people to the session and outlined the meeting structure and aims.
Evidence Brief	10	Participants were presented with an evidence brief providing the most recent information about the prevalence and disease burden of diabetes and obesity in rural and remote Australia and how this compares to other parts of the country. The evidence brief also presented information on what is known about prevention of diabetes.
Model review introduction	25	The process used to develop the maps in STICKE was described to the participants and the map presented by building the map up theme by theme. The meaning of the variables, direction and style of arrows was described to participants.
Model review	30	Participants were invited to review the A3 maps of the system relating to the causes and effects of diabetes on the IOT and place a black dot where the participant felt there was something important and a pink dot where they felt something was missing. They were offered the opportunity to augment the maps and add things they felt were missing. This provided an updated map that reflected the individual participants understanding of the system and provided data on the maps for future review.
Action review introduction	10	Using their augmented maps participants identified the places on the map where existing action was happening and wrote this on yellow sticky label and placed it on the part of the map the action was affecting, to consider where more action was needed and write this action on a blue sticky note and place this on the map where they felt it would act and to circle the areas of the map where they felt they had power and agency to act to change and reduce the prevalence of diabetes.
Action ideas and prioritize	25	Using the further developed maps, participants were then asked to consider actions that might be taken to reduce the prevalence and burden of diabetes on the IOT. These actions were described on the action ideas template and participants were asked to identify which parts of the map the action would impact.
Prioritize	15	Working in small groups of 2 or 3 people participants were then asked to share their ideas with each other and prioritize these ideas in order from highest to lowest priority. They were asked to prioritize considering both the feasibility of the action and the likely impact of the action.
Group summary to room	20	The small working groups created in the previous step reported their priority actions to the rest of the group and these actions were recorded and displayed at the front of the room.
Next steps and close	5	The next steps in the project were described and the meeting drawn to a close.

### 2.6. Ethics

Ethics approval was granted by the Deakin University Human Research Ethics Committee (project no. 2020–080). Written informed consent was obtained from all participants.

### 2.7. Research team and reflexivity

The research team for this second phase of work which focused on the causal loop exercise comprised three health economists and a population health researcher. The researchers did not have any relationships with participants and the causal loop study was undertaken remotely, via Zoom. No known biases expected.

## 3. Results

### 3.1. Phase one

Qualitative interviews in combination with the literature synthesis identified seven possible initiatives to prevent and manage diabetes in the IOT. These initiatives were tax and subsidies to improve access to fresh fruit and vegetables, sustainable local farming, engagement with community organizations and food retailers, culturally sensitive approach to care and empowering the community to co-design locally relevant diabetes initiatives.

### 3.2. Phase two

**Figure 1:**
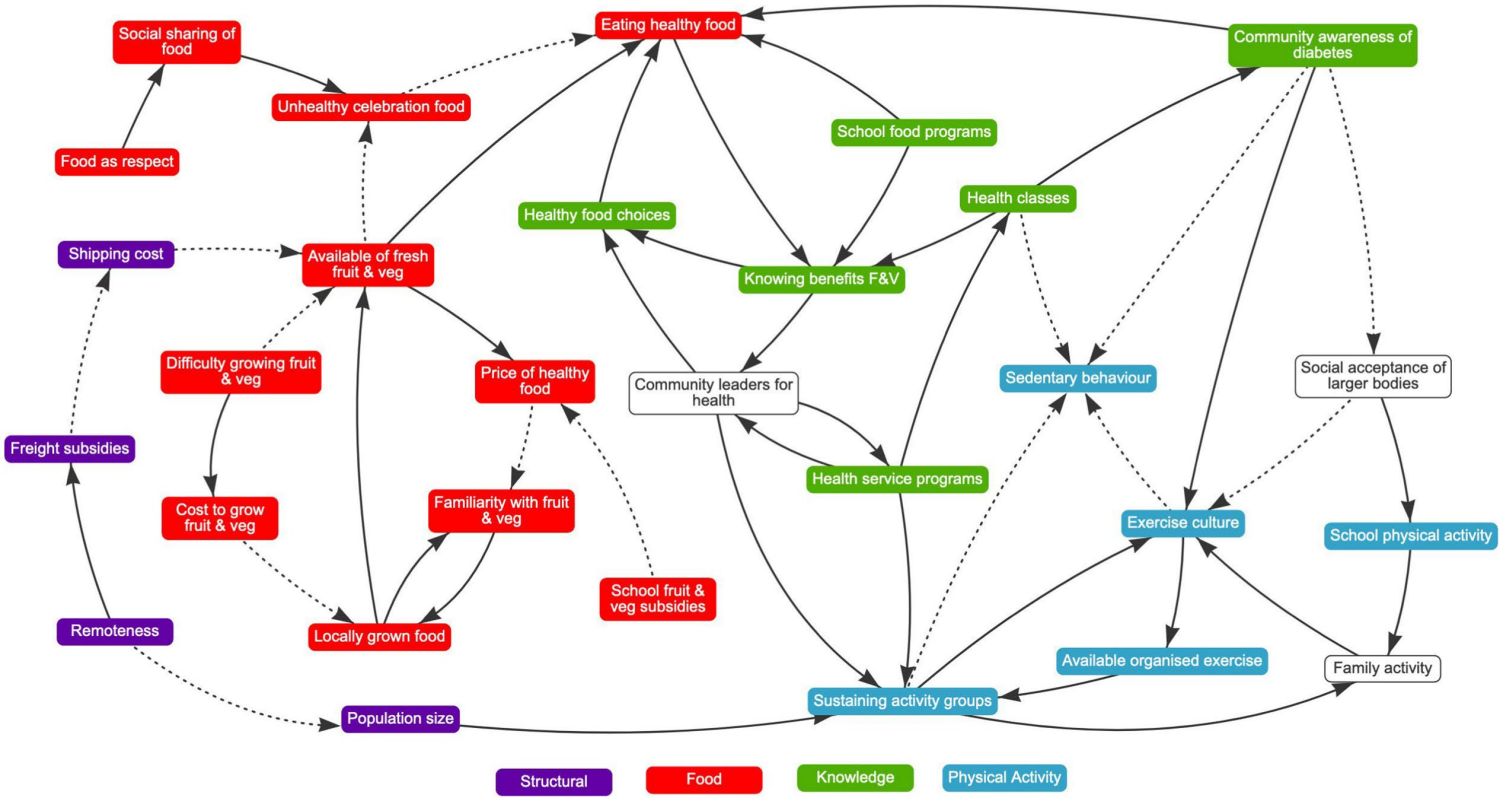
Causal loop diagram of the causes of diabetes on the Christmas and Cocos Keeling Islands.

The causal loop diagram created based on the interviews in phase one included 31 separate variables categorized into four themes (structural, food, knowledge, physical activity) (Figure 1).

Across both sessions participants identified health service programs as being valuable existing resources and price of healthy food, cost of freight, locally grown produce, community awareness of diabetes and family level activity as areas where more work could be done.

Participants across both communities identified 32 separate actions to address diabetes on the IOT (Table 2) including ten with a food focus (3 CI, 7 CKI), seven relating to physical activity (3 CI, 4 CKI), nine regarding knowledge (2 CI, 7 CKI), and six addressing structural issues (1 CI, 5 CKI).

**Table 2:** Prioritized action ideas by map theme and participating community.

Food	Physical activity	Knowledge	Structural
*CI community*			
Increase quality and availability of F&V (ahead of focus on price) Health classes on cooking Garden Co-Op – social wellbeing	Funding to broaden the types of activity available with professional support Support to overcome insurance limitations Climate adapted activity	Community education in diabetes – target community elders because of close contact Multi-generational education via the school	Formalized CO-OP to subsidize fresh produce (tax on goods arriving by air)
*CKI community*			
Portion control of lunch boxes Community based garden – accepted and new vegies Cheaper vegetables Create food markets and encourage composting (overcome the waste) Healthy menus and community leader modelling healthy foods for celebrations Cooking classes tailored to citizens Advertise healthiness of menus through food outlets	Connect health school policy – launch and engagement of parents Provides opportunity for incidental activity Female friendly exercise area (nicer environment and personalized support) Ocean pool 24/7 (safe and not tide dependent)	Community education (results of this study – accessible language) Continue with community acceptance Information for parents on food choices (social media sharing) Engage kids to reach parents Provides learning environment around F&V More relevance of fruit and veg Citizens advice bureau for food and healthy habits	External providers supporting healthy food provision (in schools) Bring key stakeholders together to help with materials Additional freight subsidy for the Islands – more freight opportunities Increase tobacco and alcohol tax and offset F&V cost Transparency on the cost of freight – a central issue which is currently less understood by participants – to support decision making

Food related priority actions ranged from increasing the quality and availability of fresh and healthy food to policies impacting portion control and lunch box contents at schools to community wide initiatives like supported community gardens, religious and diet specific cooking education and healthier menu policies in restaurants and schools. Physical activity actions included those to adapt to the local climate: notably heat and humidity and provision of experts in physical activity to provide detailed advice for the diverse members to the community (specific religious groups with clothing constraints, women only activities, older adult, transient workers) and support to overcome barriers imposed by cost of insurance for physical activity programs. Structural changes identified included developing relationships with external providers to improve health of food in schools, subsidizing freight and taxing tobacco and unhealthy foods into the islands to address the cost of healthy fruit and vegetables.

## 4. Discussion

### 4.1. Main findings

The community members of the Christmas and Cocos Keeling Islands provided in depth description of the causes of diabetes and obesity and the context specific issues that affect the rates of these diseases and hamper efforts to prevent them. These factors included issues around freight costs and frequency affecting availability and prices of fresh fruits and vegetables, quality of soil and need for facilitation of community gardening, community awareness of diabetes and sustainability of activity groups due to low population size and transient workforce, and an exercise culture. The communities were able to engage with a systems map of these causes and develop evidence informed priorities for action across multiple systems on the islands and at multiple levels of intervention.

There were multiple examples of proposed actions reaching across the themes and no examples that shifted the emphasis on individual decision making, rather placing the emphasis on policy and environment change to make the healthier choice the easy choice. These actions included health classes on cooking, community-based gardening, sporting facilities that suit local conditions such as climate-adapted facilities and ocean pools (Table 2). Similar outcomes have been seen in whole of community efforts to prevent obesity where the levels of engagement and the focus on system rather than individual are considered critical aspects to the approach (19).

The research presented here has enabled the IOT community to respond to several of the directions described for obesity prevention in the wider literature. For example, these findings echo the direction from the Lancet Commission on Obesity to consider the broader environmental system in the development of health-related actions, where IOT have shown clear understanding of the need for change at the intersection of health-related behaviors, outcomes like diabetes and obesity ad climate change (16). Through group model building the community has identified factors like population growth, transport costs, freight impacts and stigma as they relate to healthy food choice and physical activity. These results echo similar findings from studies in mainland Australia (19) where applying methods that empower communities to engage with complexity leads to more nuanced and deep understanding of the intersections between climate, community leadership and health (15).

### 4.2. Strengths

The shift in the literature from individual victim blaming for poor decision making to acknowledging and trying to address environmental determinants represents one of the commonly acknowledged strengths of the use of group model building techniques (28). This ability to locate initiatives between systemic and systematic efforts also lends itself to policy development (29). A particular strength of the method is the participants are responding to a detailed evidence brief to confirm a model that represents the complexity of the cause and effect of diabetes and obesity. The use of methods from system dynamics results in information on the dynamic nature of these relationships and how each of these key factors interact and change over time to lead to the observed problem. In the current study the identification of taxation of foods creating diabetes and obesity whereby resulting income is directed toward subsidies healthier behaviors provides a salient example.

The use of co-design principles provided new insights and deeper engagement than more traditional approaches to intervention design. The co-design approach is particularly relevant for the Christmas and Cocos (Keeling) Islands because they represent a remote and culturally diverse group of communities. These challenges mean the conditions in which diabetes related behaviors and policy occur are different to those in major cities. Co-design actively works with communities to understand what interventions will be suited to the unique local conditions and how known evidence-based interventions need to be adapted to be effective in unique communities like those represented in this study. These approaches have also shown to be empowering for communities who are not usually actively engaged in developing changes to improve health (e.g., 11, 12) and for remote and rural communities co-design has been seen to positively impact usually intractable problems like child obesity (20). Similarly, the multi-phase approach to the study meant data were able to be considered, synthesized and fed into subsequent steps, demonstrating to the community the respect for which their data was held and the utility it had for supporting their efforts to improve health on the islands.

### 4.3. Limitations

It is widely acknowledged that participatory processes are more powerful when conducted in person and due to COVID and State border closures the research team were unable to physically attend sessions on the islands. The use of videoconferencing is emerging in this type of research and this project represents one of the first to present a hybrid model of activities in place (via Zoom) and data collection and synthesis remotely. Previous research into the effectiveness of participatory methods has emphasized the importance of meeting face to face, and this may be amplified in marginalized populations. Research with Aboriginal populations, for example (12) suggest face to face ensures more collective group work collectively, better sharing and alignment of aims and agendas, and important non-verbal communication. Because of these factors the lack of face-to-face engagement may limit the potential power of, and buy-in to, the solutions generated by the participants and full understanding of the ITO context is less likely. While in this study participants were together in one room, the facilitators dialed in from a remote location suggesting these issues are likely to impact the study outcomes. While not ideal, it may represent an incremental step in making such methods available for remote communities that prior to high quality videoconferencing facilities, was unavailable.

### 4.4. Implications for practice

It is clear there is desire and will on the islands for change to prevent chronic disease, in this case diabetes and obesity. These efforts also have the potential to positively impact mental health on the islands and the majority of actions are translatable to policy positions, supported by community members suggesting the islands are likely to be receptive to interventions.

The purpose of this research was to develop a map of the causes of obesity and diabetes in the Indian Ocean Territories (IOT), evaluate the effectiveness of a facilitated group model building process and develop a greater understanding of the causes of obesity and diabetes within the IOT.

The facilitated group model building process identified a list of actionable options which serve as a starting point for a community response to the problem. Participants prioritized these action ideas based on potential impact and feasibility, with emphasis on whether changes were single actions or engaged across several variable or engaged feedback loops.

New trials that build on the community engagement and good will generated here would enable insight into whether these actions can be translated into practical policy which have the potential to be implemented on the islands and what effect these may have on diabetes and obesity.

These efforts also have the potential to positively impact mental health on the islands and the majority of actions are translatable to policy positions, supported by community members suggesting the islands are likely to be receptive to interventions.

### 4.5. Future research

New trials that build on the community engagement and good will generated here would enable insight into whether these actions can be translated into practical policy which have the potential to be implemented on the islands and what effect these may have on diabetes and obesity. Previous examples using these techniques in community wide intervention design have proven effective and have become embedded as policy positions in multiple jurisdictions (e.g., (30, 31)). Using methods to track and adapt these trials in real time is a further area for advance and emerging techniques provide the utility to achieve these aims (32).

## 5. Conclusion

Diabetes and obesity are major health concerns for the Christmas and Cocos (Keeling) Islands. These concerns are exacerbated by remoteness of the islands and through co-design new ways to tackle these have been identified which are acceptable and of high priority to the community and which appear to have policy relevance.

## Data availability statement

The raw data supporting the conclusions of this article will be made available by the authors, without undue reservation.

## Ethics statement

The studies involving human participants were reviewed and approved by Ethics approval was granted by the Deakin University Human Research Ethics Committee (project no. 2020–080) and the Aboriginal Health and Medical Research Council Ethics Committee (project no. 1692/20). Written informed consent was obtained from all participants. The patients/participants provided their written informed consent to participate in this study.

## Author contributions

SA and EL conceived and developed the initial research idea. SA and SM contributed to the writing of the manuscript and analyzed these interview data. SM, SB, and EL conducted the qualitative interviews. SA, SM, and SB designed and conducted the systems mapping workshops. EL supervised the project. All authors contributed to the article and approved the submitted version.

## Funding

This research is funded by the Public Policy and Societal Impact Hub at ANU under the Greenhouse program.

## Conflict of interest

The authors declare that the research was conducted in the absence of any commercial or financial relationships that could be construed as a potential conflict of interest.

## Publisher’s note

All claims expressed in this article are solely those of the authors and do not necessarily represent those of their affiliated organizations, or those of the publisher, the editors and the reviewers. Any product that may be evaluated in this article, or claim that may be made by its manufacturer, is not guaranteed or endorsed by the publisher.

## Footnotes

^1^Systems Thinking in Community Knowledge Exchange (STICKE) is a cloud-based software platform that supports a “systems thinking” approach to tackling complex problems. STICKE was developed by researchers at Deakin University in collaboration with Victorian communities. The software guides users through the creation of a system “map” that can then be used to help explore the problem and potential intervention points. STICKE can support communities in mapping a complex problem and its drivers at all levels of policy and decision-making authority and identify appropriate and feasible locally led responses.
